# The Direction of Symptoms

**Published:** 1888-08

**Authors:** 


### THE DIRECTION OF SYMPTOMS.

Professor Kent says: "In every case of disease you should carefully notice the *direction* of symptoms. In acute diseases which run their course without treatment, the same succession of symptoms may always be observed, viz.: the symptoms which first appear are the last to disappear. If the symptoms be carefully watched throughout the course of the disease, they will be seen to disappear in the reverse order of their coming. But if the disease is interfered with by treatment, the natural course of the disease will not be followed. But if the proper remedy is administered, and in the proper manner, it will check the progress of the disease in any stage, causing a disappearance of all the symptoms. In chronic diseases especially is this knowledge of the direction of symptoms most valuable. In all diseases—more easily observed in the chronic—when being cured homœopathically, symptoms disappear from within outward, from above downward, and in the reverse order of their coming. Thus, in rheumatism of the shoulders, after administering the appropriate remedy, the disease may go to the hips, afterward to the knees. Then you may be sure, although your patient suffers more pain than before, that he is getting well. On the other hand, if the disease is incurable or is getting worse, the direction of the symptoms will be reversed. They will travel from below upward and from without inward. For instance, if a patient has rheumatism of the shoulders, as in the previous case, and you administer the indicated remedy, if the disease is incurable it will be very likely to attack the heart or go to some other vital part."—*Medical Era.*

